# Content of lunchboxes of Dutch primary school children and their perceptions of alternative healthy school lunch concepts

**DOI:** 10.1017/S1368980022002282

**Published:** 2023-03

**Authors:** Frédérique C Rongen, Ellen van Kleef, Monique H Vingerhoeds, Jacob C Seidell, Sanne Coosje Dijkstra

**Affiliations:** 1 Department of Health Sciences, Faculty of Science, Vrije Universiteit Amsterdam, Amsterdam Public Health Research Institute, De Boelelaan 1085, Amsterdam 1081 HV, the Netherlands; 2 Marketing and Consumer Behaviour Group, Wageningen University, Wageningen, the Netherlands; 3 Food, Health & Consumer Research group, Wageningen Food & Biobased Research, Wageningen, the Netherlands

**Keywords:** School lunch, Lunchboxes, Child, Schools, Diet, Feeding behaviour

## Abstract

**Objective:**

To investigate the content of lunchboxes of primary school children and to examine children’s support and preferences for alternative healthy school lunch concepts.

**Design:**

A cross-sectional study among Dutch children from seven primary schools. The content of the lunchboxes was assessed by photographs. Support and preferences for alternative lunch concepts were examined via a self-reported questionnaire. Linear regression analyses were used to investigate the associations between children’s support and preferences and sex, educational group and migration background.

**Setting:**

The Netherlands.

**Participants:**

Primary school children.

**Results:**

A total of 660 children were included (average 9·9 years old). Most lunchboxes contained sandwiches and a drink. Few lunchboxes contained fruit or vegetables. The alternative school lunch concepts elicited mixed support among children. The lunch concepts ‘Sandwiches prepared by the children themselves’ and a ‘hot lunch buffet’ had the highest mean support, while the concept ‘a healthy lunch brought from home’ was the most preferred concept. Small significant differences were observed depending on sex, educational group and migration background.

**Conclusion:**

Lunchboxes of Dutch children contained sandwiches and a drink but rarely fruit and vegetables. Among different alternatives, children reported the highest support for the preparation of their own sandwiches in class or a hot lunch buffet. Future studies should investigate if these alternative lunch concepts improve the dietary intake of children.

Children in most Western countries often do not meet the recommended dietary guidelines that increases their risk of diet-related chronic diseases as obesity^(1)^. This is also the case in the Netherlands where most children eat and drink too many snacks and drinks high in sugar and consume too much fat and salt^(2)^. Taking into account that the eating habits of children established in childhood track into later life, it is important to teach children healthy eating habits early on^(3,4)^.

A healthy diet for children can be stimulated in various settings, for example in the home, neighbourhood and school environment. From the age of 4, Dutch children spend a large amount of their time at school^(5)^. Therefore, public health interventions organised in school settings may contribute to healthy dietary habits. In addition, school interventions have the potential to reach children from all socio-economic backgrounds and therefore reduce the observed socio-economic inequalities in dietary intake^(6,7)^. Effective methods for schools to promote a healthier diet for children are nutrition education, rules for healthier foods and drinks or offering a healthy school-provided meals^(8)^. A study performed in the Netherlands on differences between the content of home packed lunches brought to school compared with consuming lunch at home showed that children who brought a home-packed lunch to school had a higher intake of sugar-sweetened beverages (defined as carbonated soft drinks, other non-carbonated sugar-sweetened drinks (water-based beverages that contain sugar) and sport drinks^(9)^) during lunch than those who ate their lunch at home^(10)^. However, both lunches consumed at school and at home contained a very low quantity of fruit and vegetables^(10)^. Other studies also showed that lunchboxes contained large amounts of energy-dense foods and drinks high in sugar^(11,12)^. However, most information about the content of the lunch at schools is via self-reported data of the parents or children and an objective measurement is lacking.

School-provided lunches have proven to be effective in improving children’s diets and often have a better nutritional quality than packed lunches brought from home^(3,13–19)^. A study among Danish children showed improvement in the overall dietary intake when their packed lunches were replaced by a school-provided meal for a period of 3 months. Children participating in this school lunch program consumed half the amount of sugar, Na and saturated fat during lunch compared with those taking a packed lunch to school. Furthermore, most children participating in the school lunch program ate vegetables while this was only 8 % of the children who brought a packed lunch^(8)^. A study in Norway showed that serving a free school meal increased children’s intake of healthy foods for a period of a year^(20)^, and a study in Sweden showed that healthy school lunches have a positive contribution to intake of children^(21)^.

In contrast to many other countries, primary schools in the Netherlands do not serve school-provided lunches. Not so long ago most Dutch children either eat their lunch at home and had a more traditional timetable in which there was a morning and an afternoon schedule in school, separated by a long lunch break of 60 to 90 min during which children could go home to eat lunch. However, over the past few years, an increasing number of schools has shifted towards a continuous timetable, which means that all school days will last from morning to halfway the afternoon with a short 15 min lunch break where all children eat their packed lunch from home at school. This transition, which makes that more Dutch children eat their lunch at school, creates an opportunity for the introduction of a healthy school-provided lunch.

When developing a healthy school-provided lunch program from scratch, understanding children’s preferences and support for alternative school lunch concepts is crucial for the acceptance of such a considerable change. Research has shown that children are able to express their views when it comes to their food choice^(22)^. Our recent qualitative study showed that Dutch children, parents and school staff are open towards the idea of a school-provided healthy lunch. To smooth a potential transition to a new and unfamiliar lunch situation, it is important to have a better insight in the content of children’s current lunch and their support for a set of alternative school lunch concepts. This support can be critical for implementation and can increase the acceptance of such changes. To our knowledge, no research has previously been done on children’s specific preferences and support regarding a healthy school lunch and alternative school lunch concepts in the Netherlands. Also outside the Dutch setting, understanding and involving children in such major potential changes is relevant in order to increase the chance of success of school meal programs and adapt school meal programs to the needs and preferences of children. Therefore, the aim of this study is twofold. First, we investigated the content of the school lunchboxes of Dutch primary school children. Second, we examined children’s support and preferences for six healthy school lunch concepts varying in type of food served and presentation mode. Besides, we examined whether the lunch content and the support and preferences varied depending on sex, educational group and migration background of the children.

## Methods

### Design and procedure

This study is part of the Healthy School Lunch project in the Netherlands^(23)^. The overall aim of this project is to encourage healthy eating behaviour of children at primary schools by offering a healthy school lunch, based on the Dutch guidelines for a healthy diet^(24)^. In this part a cross-sectional study design was used to examine the content of children’s lunchboxes and their support and preferences for different healthy school lunch concepts in primary schools in two cities in the Netherlands including Amsterdam with approximately 854 000 habitants and Ede, with approximately 115 000 habitants. Data were collected between September and November 2017. The study was approved by the Social Ethical Commission of Wageningen University Research, the Netherlands. Most schools in Amsterdam (206 of 221) as well as schools in Ede with a known interest in nutrition were approached by email to inform them about the study. Follow-up phone calls were made until a sample of different schools (e.g. size and neighbourhood) participated. The following selection criteria were used for the inclusion of the schools: (1) schools in primary education; (2) schools in different neighbourhoods and (3) schools with a lower or higher social economic position population. The level of social economic position was based on the social economic position of the neighbourhood where the school was located and is derived from a number of characteristics such as the people who live there, their educational level and income, which is defined by the local central office for statistics^(25)^. Seven schools (three in Amsterdam and four in Ede) agreed to participate, and these schools distributed a letter among parents, with information about the study. Parents had the opportunity to refuse participation of their child via passive informed consent. Children also had the opportunity to refuse participation before or during the data collection. Data collection took approximately 30 min, and all children received a small present for their participation. The sample consisted of 720 children, of which twenty-two parents refused participation of their child and thirty-eight children were absent during the day of measurements (e.g. sickness). In total, 660 children participated in the study.

### School lunchbox content assessment

The content of the lunchbox was assessed via a photo of each lunchbox and by the use of code cards. All children consumed their home-packed lunch in their own classroom. At the beginning of the lunch break, children were asked to take their lunchboxes and to present the content of their lunchbox (foods and drinks) that they were planning to consume during lunch on their table. Each lunchbox was provided with a code card that contained an ID number of the child and questions about the number of slices of bread, the type of bread and toppings, the type and quantity of drinks and other products. Children were asked to postpone eating until the researcher filled out the code cards and took a picture of the content of the lunchbox. There were two or three researchers per class room, one was filling out the code cards and one took pictures of the lunchboxes.

Each photo and corresponding code card was entered in a data entry file that was made using the Qualtrics survey tool^(26)^. All foods and drinks and their quantity on the code cards were entered. In case it was not observable what the specific product was (e.g. full-fat or low-fat milk), the most commonly brought product version in the Netherlands was entered in the database. The content of the lunchboxes was classified into food groups that correspond to the most commonly brought lunch products. The following food groups were created: bread; white, brown/whole grain and other (e.g. cornbread, croissant, currant bun, unclear), sandwich toppings; processed meats, peanut butter/nut paste, cheese and cheese products, sweet bread toppings (e.g. chocolate sprinkles or jam), hummus/sandwich spread/sandwich salad toppings, no topping, vegetables and other (e.g. fish and eggs), drinks; water/tea, sweetened drinks and unsweetened drinks (including milk, butter milk (a common unsweetened fermented low fat (0·9 g/100 ml) dairy drink in the Netherlands), fruit, vegetables and other foods (e.g. nuts, wraps, pancakes, cookies, chocolate, slices of sausage, donuts and chips).

Parents were not informed on which day photos would be taken of the lunch boxes of their children in order to avoid that parents would pack a healthier lunch on the day that the researchers were visiting the school. Due to a miscommunication between the researchers and one of the schools in Ede, one data collection day occurred when children had a short school schedule (till 12:00 p.m.) and did not bring lunch to school. Therefore no photos were taken of their lunchboxes (*n* 25). Two schools had a traditional time schedule where children have the choice to stay over at school or go home for lunch. Children who went home had no lunchboxes and therefore no photos were taken (*n* 137). Eleven children refused a photo of their lunch box. These children did filled out the questionnaire about the perceptions of the healthy school lunch concepts. In total, 487 photos of lunchboxes were made.

### Evaluation of the alternative healthy school lunch concepts

The alternative healthy school lunch concepts were evaluated by the use of two questions. The first question examined the support for each concept individual, and the second question examined the preference for the best concept. The evaluation of the six alternative healthy school lunch concepts was measured with a digital questionnaire that was filled out on a tablet with a Qualtrics app, which took children on average 15 min. This approach was pre-tested in a small group of children. Based on the interviews and the preferences of children and parents in our previous study (Frédérique C Rongen, S Coosje Dijkstra, Tobie H Hupkens, Monique H Vingerhoeds, Jaap C Seidell and Ellen van Kleef, unpublished results), six alternative school lunch concepts were developed. School lunch concepts were described in terms of the food and drinks offered in general, the way it was served and whether children had a free choice (for description and pictures of the six lunch concepts see online supplemental 1). All the concepts were based on the Dutch dietary guidelines and in accordance with the Dutch Nutrition Centre^(24)^. The six developed school lunch concepts were (1) a healthy lunch brought from home; (2) packed sandwiches provided at school; (3) sandwiches prepared by children themselves at school; (4) soup or salad with bread provided at school; (5) a hot lunch on plates provided at school and (6) a hot lunch buffet provided at school. Before introducing the concepts, it was explained that the following aspects were the same for each lunch concept: every child gets the same food, the provided beverages will be water, milk or buttermilk and that allergies and special diets (e.g. halal and/or vegetarian) were taken into account. Furthermore, it was stated that the children consumed their lunch into their own classroom with their classmates, that they were given enough time to eat (approximately around 30 min) and that the food provided in each lunch concept would be slightly different every day of the week. Concepts were randomly displayed for each child and after each concept children could state their support by answering the question ‘how much do you like this concept if this was your lunch at school every day?’. Children could select their support by choosing one of the five smileys. The smileys ranged from a red smiley, orange smiley, yellow smiley, light green smiley and a dark green smiley, faces ranging from sad to happy. Literature showed that especially the use of smiley rating questions is child-friendly and makes children feel at ease when filling out questionnaires (W Yahaya and S Salam, unpublished results). Preference for the alternative school lunch concepts was assessed with a final question ‘which concept do you prefer the most to have at your school?’. Children could select their preference by selecting one concept.

### Demographic variables

Information on age, sex and education group of the children was asked at the end of the questionnaire. Migration background was obtained with three open-ended questions in which was asked in which country they, their mother and their father were born. Children were categorised as having a migration background when at least one of their parents was not born in the Netherlands^(27)^. Migration background is further categorised into no migration background, Western or Non-Western. Children were categorised as having a no migration background or a Western migration background if they had no migration background or if they were born in a country in Europe, North-America or Oceania. Non-Western migration background included countries of origin as Africa, Latin-America or Asia.

### Place of consumption

Schools with a continuous schedule or a traditional schedule were included in this study. To define the place of consumption, all children with a continuous schedule were categorised as consuming their lunch at school. Children with a traditional schedule had the opportunity to go home or to stay at school for lunch received an extra question ‘where do you consume your lunch today?’.

### Analysis

Descriptive statistics were used to summarise the characteristics of the study sample. The content of the lunchboxes per food group was presented in means, standard deviations (sd) and percentage of users. Logistic regression models were used to investigate the association between the presence of brown/whole grain bread, white bread, water/tea and sweetened drinks and sex, migration background and educational group. Analyses were (if possible) adjusted for sex, educational group and migration background. OR and their 95 % CI were presented.

To analyse the difference in children’s support for the different alternative healthy school lunch concepts by sex, migration background and educational group, a linear regression is performed. Analyses were adjusted for sex, educational group and migration background. Regression coefficients (*β*) and their 95 % CI were presented. In the supplements, an overview of the percentages per smiley for each school lunch concept divided by sex, educational group and migration background was added. This was done to check if the results of the regression analyses were confirmed since there is discussion about the use of Likert scales as a continuous variable^(28,29)^. To analyse the difference in children’s preference for one of the alternative healthy school lunch concept by sex, migration background and educational group logistic regression models were used. R and their 95 % CI were presented. Data were analysed with IBM SPSS Statistics 24^(30)^.

## Results

### Characteristics

The sample included 660 children, of which 296 boys and 343 girls (Table 1). They were on average 9·9 years (sd = 1·2) old. More than half of the children were in educational groups 7 and 8 (58·6 %) (comparable with US elementary school grades 5 and 6) and had no or a Western migration background (67·0 %).

**Table 1:** Characteristics of the sample

		Total (*n* 660)	
		*n*	%
Sex	Male	296	52·0
	Female	343	44·8
	Missing	21	3·2
Educational group	5–6	252	38·2
	7–8	387	58·6
	Missing	21	3·2
Migration background	No migration background	403	61·1
	Western migration background	39	5·9
	Non-Western migration background	182	27·6
	Unknown/missing	36	5·5
Place of lunch consumption	Home	157	23·8
	School 30 min break	159	24·1
	School 15 min break	344	52·1
Photograph of the lunch box	Yes	487	73·8
	No	173	26·2

### Lunch box content assessment

In total, 487 lunchboxes were photographed (Table 2). The majority of the children brought brown or whole grain bread (70·2 %) for lunch with an average of 2·3 (sd = 0·7) slices of bread per lunchbox, followed by white bread (18·9 %) with an average of 2·6 (sd = 1·0) slices of bread per lunchbox. Most children had processed meats (44·4 %), peanut butter (38·8 %) or cheese products (29·2 %) as a sandwich topping. Almost half of the children drink water or tea (42·9 %) during their lunch with an average estimated amount of 400 ml (sd = 100) per drink. The other half of the children brought sweetened drinks (42·9 %) during lunch with an estimated average amount of 270 ml (sd = 100) per drink. Only 5 % of the lunchboxes contained fruit and 6 % of the lunchboxes contained vegetables.

**Table 2:** Content of the lunchboxes per food group (*n* total lunchboxes = 487)

	*n*	%	Mean	sd
Bread (slices)				
Brown/whole grain only	342	70·2	2·3	0·7
White only	92	18·9	2·6	1·0
Other only	17	3·5	2·0	1·0
Combination white and brown	9	1·9	2·8	0·7
Combination white or brown with other	1	0·2	4·0	
Sandwich spreads or toppings (unit)				
Processed meats	216	44·4	1·6	0·7
Peanut butter/nut paste	189	38·8	1·6	1·0
Cheese and cheese products	142	29·2	1·5	0·6
Sweet bread toppings	128	26·3	1·4	0·6
Other	21	4·3	1·5	0·5
Hummus/sandwich spread/sandwich salad toppings	18	3·7	1·5	0·5
No topping	14	2·9	1·1	0·4
Vegetables	10	2·1	1·5	0·5
Drinks (ml)				
Water/tea only	209	42·9	400	100
Sweetened drinks only	209	42·9	270	100
Other unsweetened drinks only	20	4·1	230	50
Combination	7	1·4	600	150
Fruit (piece)	25	5·1	1	0·3
Vegetables (piece)	29	6·0	1·1	0·4
Other foods (piece)	43	8·8	1·1	0·3

ml, millilitre.

Table 3 shows the adjusted associations between sex, educational groups and migration background and the presence of brown/whole grain bread, white bread, water/tea and sweetened drinks in the lunchbox. The results showed that boys were more likely to bring sweetened drinks (OR 1·42, 95 % CI (1·00, 2·00)), but were less likely to bring white bread (OR 0·58, 95 % CI (0·35, 0·95)) for lunch than girls. Children from educational groups 5 and 6 were less likely to bring white bread (OR 0·57, 95 % CI (0·35, 0·92)) than children from educational groups 7 and 8. Children with a non-Western migration background were less likely to bring brown/whole grain bread (OR 0·57, 95 % CI (0·40, 0·81)) and sweetened drinks (OR 0·55, 95 % CI (0·36, 0·82)) for lunch, but were more likely to bring white bread (OR 2·97, 95 % CI (1·84, 4·81)) for lunch than children with no or a Western migration background.

**Table 3:** Results of the adjusted logistic regression analyses for the associations between sex, educational groups, migration background and the content of children’s lunchboxes per food item

	Brown bread		White bread		Water or tea		Sweetened drinks	
	OR	95 % CI	OR	95 % CI	OR	95 % CI	OR	95 % CI
Sex†								
Girls (REF)	1·00		1·00		1·00		1·00	
Boys	1·09	0·79, 1·50	0·58	0·35, 0·95*	0·73	0·05, 1·02	1·42	1·00, 2·00*
Educational group‡								
Groups 5–6 (REF)	1·00		1·00		1·00		1·00	
Groups 7–8	0·87	0·63, 1·21	0·57	0·35, 0·92*	0·89	0·63, 1·25	0·53	0·53, 1·07
Migration background§								
No or a Western migration background (REF)	1·00		1·00		1·00		1·00	
Non-Western migration background	0·57	0·40, 0·81*	2·97	1·84, 4·81*	1·41	0·98, 2·03	0·55	0·36, 0·82*

Ref, reference group.

* *P* < 0·05.

† Adjusted for educational group and migration background.

‡ Adjusted for sex and migration background.

§ Adjusted for sex and educational group.

### Evaluation school lunch concepts

The mean support for the alternative healthy school lunch concepts by the children is shown in Table 4 (for frequency results of every category of the five point scale, see online supplemental 2). Generally, all the alternative concepts were positively evaluated by the children and had a mean score around or above the midpoint of the scale (score ranges from −2 to +2). The lunch concepts ‘Sandwiches prepared by the children themselves at school’ (mean = 0·54, sd = 1·21) and a ‘hot lunch buffet provided at school’ (mean = 0·49, sd = 1·39) had the highest mean support.

**Table 4:** Children’s support for the alternative healthy school lunch concepts measured on a five-point scale ranging from −2 to +2

	Mean	sd
Sandwiches prepared by the children themselves at school	0·54	1·21
A hot lunch buffet provided at school	0·49	1·39
A healthy lunch brought from home	0·44	1·21
A hot lunch on plates provided at school	0·34	1·36
Soup or salad with bread provided at school	0·25	1·34
Packed sandwiches provided at the school	0·08	1·30

Scale −2 to +2 with red (−2), orange (−1) yellow (0), light green (1), and dark green (2) smileys.

Table 5 shows the results of the adjusted association between children’s support for the healthy school lunch concepts and sex, educational group and migration background. Girls reported a higher support for the concepts ‘sandwiches prepared by the children themselves at school’ (*β* = -0·71, 95 % CI (−0·49, −0·12)) and ‘soup or salad with bread provided at school’ (*β* = -0·28, 95 % CI (−0·49, −0·07)) than boys. We observed no other statistically significant differences across sex.

**Table 5:** Results of the linear regression analyses for the associations between mean support of the alternative school lunch concepts and sex, educational group and migration background

	A healthy lunch brought from home		Packed sandwiches provided at the school		Sandwiches prepared by the children themselves at school		Soup or salad with bread provided at school		A hot lunch on plates provided at school		A hot lunch buffet provided at school	
	*β*	95 % CI	*β*	95 % CI	*β*	95 % CI	*β*	95 % CI	*β*	95 % CI	*β*	95 % CI
Sex†												
Girls	0·60		0·22		0·71		0·25		0·26		0·50	
Boys	−0·02	−0·21, 0·17	−0·18	−0·38, 0·02	−0·30	−0·49, −0·12*	−0·28	−0·49, −0·07*	−0·4	−0·35, 0·07	−0·13	−0·34, 0·09
Educational group‡												
Groups 5–6	0·60		0·22		0·71		0·25		0·26		0·50	
Groups 7–8	−0·23	−0·42, −0·04*	−0·23	−0·44, −0·03*	−0·22	−0·41, −0·03*	0·04	−0·18, 0·25	0·01	−0·21, 0·22	−0·1	−0·32, 0·12
Migration background§												
No or a Western migration background	0·60		0·22		0·71		0·25		0·26		0·50	
Non-Western migration background	0·01	−0·20, 0·22	0·32	0·10, 0·55*	0·41	0·21, 0·62*	0·38	0·15, 0·61*	0·52	0·29, 0·75*	0·41	0·17, 0·64*

*β*, beta; Ref, reference group.

* *P* < 0·05.

† Adjusted for educational group and migration background.

‡ Adjusted for sex and migration background.

§ Adjusted for sex and educational group.

Children from educational group 7 and 8 reported a lower support for the concepts ‘a healthy lunch brought from home’ (*β* = -0·60, 95 % CI (−0·42, −0·04)), ‘packed sandwiches provided at the school’ (*β* = -0·22, 95 % CI (−0·44, −0·03)) and sandwiches prepared by the children themselves at school (*β* = -0·71, 95 % CI (−0·41, −0·03)) than children from lower educational groups. We observed no other statistically significant differences across the educational groups.

Children with a Western migration background reported a higher support for the concepts ‘packed sandwiches provided at the school’ (*β* = 0·32, 95 % CI (0·10, 0·55)), ‘sandwiches prepared by the children themselves at school’ (*β* = 0·41, 95 % CI (0·21, 0·62)), ‘soup or salad with bread provided at school’ (*β* = 0·38, 95 % CI (0·15, 0·61)), ‘a hot lunch on plates provided at school’ (*β* = 0·52, 95 % CI (0·29, 0·75)) and ‘a hot lunch buffet provided at school’ (*β* = 0·41, 95 % CI (0·17, 0·64)) than children with a non-Western migration background.

The preferences for the most favourable concept are shown in Table 6. The concept ‘a healthy lunch from home had the highest preference (30·2 %), followed by ‘a hot lunch buffet provided at the school’ (26·8 %). The concept ‘packed sandwiches provided at the school’ had the lowest preference among the children (8·8 %).

**Table 6:** Children’s preference for the best healthy school lunch concept

	%
A healthy lunch brought from home	30·2
A hot lunch buffet provided at school	26·8
Sandwiches prepared by the children themselves at school	13·8
Soup or salad with bread provided at school	10·8
A hot lunch on plates provided at school	9·7
Packed sandwiches provided at the school	8·8

Table 7 shows the associations between sex, educational groups and migration background and the preference for the most favourable lunch concepts. The results showed that children from educational groups 7 and 8 had a lower preference for the concepts ‘a healthy lunch brought from home (OR 0·70, 95 % CI (0·49, 0·99)) than children from educational groups 5 and 6. Children with a non-Western migration background had a lower preference for the concept ‘a healthy lunch from home (OR 0·53, 95 % CI (0·35, 0·80)) and a higher preference for the concept ‘A hot lunch on plates provided at school’ (OR 2·15, 95 % CI (1·26, 3·67)) than children with no or a Western migration background. There were no significant differences between sex and the preference for each lunch concept. Results of the ordinal logistic regression analysis confirmed all the results, see online supplemental 3.

**Table 7:** Results of the adjusted logistic regression analyses for the associations between sex, educational groups, migration background and preferences for the most favourable lunch concept

	A healthy lunch brought from home		Packed sandwiches provided at the school		Sandwiches prepared by the children themselves at school		Soup or salad with bread provided at school		A hot lunch on plates provided at school		A hot lunch buffet provided at school	
	OR	95 % CI	OR	95 % CI	OR	95 % CI	OR	95 % CI	OR	95 % CI	OR	95 % CI
Sex†												
Girls (REF)	1·00		1·00		1·00		1·00		1·00		1·00	
Boys	1·09	0·77, 1·54	0·90	0·90, 2·74	0·72	0·45, 1·15	0·81	0·48, 1·35	1·56	0·92, 2·66	0·83	0·58, 1·19
Educational group‡												
Groups 5–6 (REF)	1·00		1·00		1·00		1·00		1·00		1·00	
Groups 7–8	0·70	0·49, 0·99*	0·59	0·34, 1·03	1·07	0·67, 1·72	1·58	0·91, 2·74	1·25	0·72, 2·18	1·30	0·90, 1·89
Migration background§												
No or a Western migration background (REF)	1·00		1·00		1·00		1·00		1·00		1·00	
Non-Western migration background	0·53	0·35, 0·80*	1·11	0·61, 2·04	0·67	0·39, 1·16	1·39	0·81, 2·36	2·15	1·26, 3·67*	1·29	0·88, 1·89

Ref, reference group.

* *P* < 0·05.

† Adjusted for educational group and migration background.

‡ Adjusted for sex and migration background.

§ Adjusted for sex and educational group.

## Discussion

The first objective of this study was to identify the content of the lunchboxes of Dutch primary school children and investigate potential differences between sex, migration background and educational group. The results showed that children’s lunchboxes contain a traditional Dutch lunch with mostly brown, whole grain or multigrain bread with either cheese, processed meat or sweet bread toppings, with low quantities of fruits and vegetables. The majority of the drinks children brought from home were water or sweetened beverages. These results are consistent with the results from the Dutch National Food Consumption Surveys, which also showed that lunches of primary school children mostly contain bread^(31)^. Other studies confirmed that the consumption of sweetened beverages among children is high^(32)^.

Only a few of children’s lunchboxes in this study contained fruit or vegetables, which is worrisome given the low intake of children’s fruit and vegetables worldwide^(33)^. This result may be explained by the fact that children may already have eaten fruit during the mid-morning school break. The Dutch National Food Consumption Surveys showed that children mostly ate fruit in between meals and not during lunch. A possible explanation for the low consumption of vegetables in this study might be that in the Dutch National Food Consumption Surveys, it was found that usually children only eat vegetables during dinner and not during lunch^(2,31)^. In a qualitative study with Dutch and Flemish children, children indicated that they rarely brought vegetables to school for lunch, and it was perceived as weird to eat vegetables at school^(34)^. Considering the low amount of fruit and vegetables consumption in primary schoolchildren, it is important to find ways to increase their intake during lunch.

The second objective was to examine the support and preferences of children for alternative school lunch concepts and whether this differed across sex, migration background and educational group. Most children reported a neutral or positive support towards the alternative school lunch concepts, but they reported the most support for the healthy school lunch concept ‘Prepare your own sandwiches’ and ‘a hot lunch buffet provided at school’ and had the highest preference for the lunch concept ‘a healthy lunch brought from home’. This finding can be explained by the fact that it is possible that many children like to keep their lunch familiar, but that they also have the possibility to choose what they consume for lunch^(22)^. Besides, the fact that children choose the option that is most familiar can also be explained by the fact that it could be difficult for children to evaluate lunch concepts they have never experienced before. It takes time to appreciate and learn about new ways of consuming lunch at school. From our results, there are some small differences between sex and educational group and migration background.

### Strengths and limitations

A strength of this study was the relatively large sample size of 639 children who filled out the questionnaire and 487 photographs of children’s lunchboxes. Moreover, the schools participating in this study are from two different cities, which represents a large city (around 854 000 inhabitants) and a small city (around 115 000 inhabitants). Instead of using self-reporting questionnaires to determine the content of children’s lunches, we used photographs, which gives a more objective view and is not prone to possible socially desirable answers or recall bias. Besides, for young children, it is not possible to describe the content of their lunchboxes in detail which makes photographs a better suited method. Furthermore, to our knowledge, this is the first study that investigated the support and preferences of alternative school lunch concepts.

Several limitations of this study should also be considered. First, it should be noted that only primary schools in the cities Ede and Amsterdam were included. The content of children’s lunchboxes at schools in villages or rural areas could differ from what is observed in this population. Furthermore, the photographs of the lunchboxes used in this study were only taken during one particular school day. Therefore, it is possible that children could have had a healthier/unhealthier lunch on the other days of the week. However, due to time and cost constraints, it was not possible to examine the content of the lunchbox on multiple days. Furthermore, the food or drinks displayed in the photographs could not be categorised by type or brand. Besides, there were no photos taken after the lunch was consumed, which gives only an indications of what they brought to school and not what was actually consumed. Finally, the statistical testing of differences between school lunch concepts evaluations was performed with a linear regression what may lead to an underestimation of the results. However, the ordinal logistic regression analysis showed similar results.

Based on the results of this study, we have several recommendations for research and practice. This study showed that the current lunch of primary school children leaves substantial room for nutritional improvement. This can be done through several actions including for example school food policies (e.g. no sugar-sweetened beverages and more fruit and vegetables) or by providing a healthy school lunch. School food policies regarding a healthy lunch have been shown to only moderately impact the nutritional quality of lunchboxes^(35)^. Providing a national school meal program showed more positive results^(3,13–17)^. For countries such as the Netherlands, where there is currently no national school meal program in place, more research about the possibilities of implementation is needed. Our results showed that children reported the highest support for a concept most familiar to their current lunch situation. However, before implementing a particular school lunch concept, it is necessary to investigate the support of other stakeholders including the parents and schools since it is important for successful implementation to have support from all the stakeholders involved (e.g. children, parents and schools).

## Conclusion

The purpose of this study was to gain insight into the current content of lunchboxes that contained mostly a traditional Dutch lunch with bread, a drink and little fruit or vegetables. This leaves room for nutritional improvement. If a healthy school lunch provision will be implemented, children have the highest support and preferences for a concept that is the most familiar with the current situation. Children in this study had the highest support for the preparation of their own sandwiches in class and the highest preference for a healthy lunch from home. More work integrating insights from this study into the development of a school lunch program and studies towards the effectiveness of a school lunch provision is needed.

## Supplementary material

For supplementary material accompanying this paper visit https://doi.org/10.1017/S1368980022002282. click here to view supplementary material
