# Supplementary material for: Content of lunchboxes of Dutch primary school children and their perceptions of alternative healthy school lunch concepts
Source: Public Health Nutr. 2022 Oct 21;26(3):554–62. doi: 10.1017/S1368980022002282 (PMC9989704; doi:10.1017/S1368980022002282)
Supplement: Supplementary file 1 [file S1368980022002282sup001.docx]

**Supplemental 1 Presentation of six lunch concepts with text and two pictures.**

| Concept | Description | Picture 1 | Picture 2 |
| --- | --- | --- | --- |
| a healthy lunch brought from home | Children bring their own lunch from home every day. The school makes strict agreements with the parents about what is and what is not healthy to bring to school. | 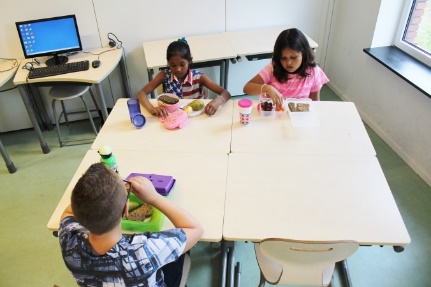 | 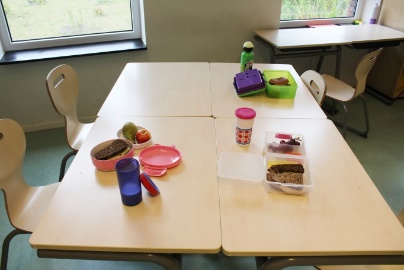 |
| packed sandwiches provided at the school | Each child will get every day during lunchbreak a ready-to-eat packed lunch with sandwiches, a bun or a wrap. This packed lunch also includes fruit or vegetables. | 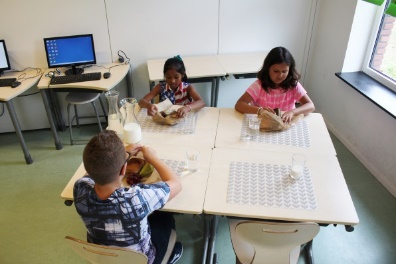 | 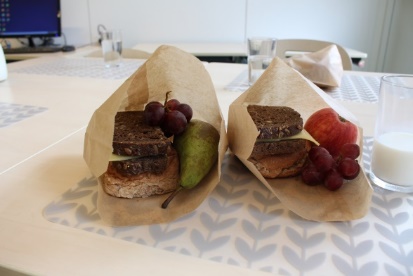 |
| sandwiches prepared by the children themselves at school | Every day during lunchbreak bread and spreads or toppings are provided by the school. They also provide fruit, vegetables and something extra like an egg. The lunch will be prepared by the children at their own table. | 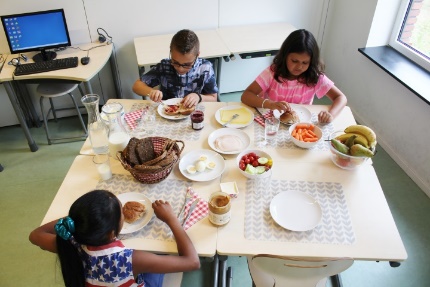 | 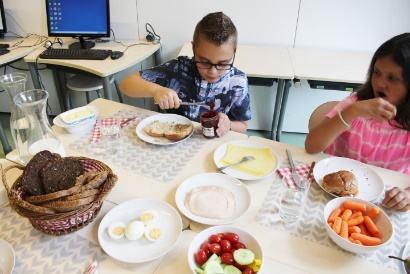 |
| Soup or salad with bread provided at school | Every day during lunchbreak children get either soup with sandwiches or salad with sandwiches provided by the school. Vegetable snacks are also available. | 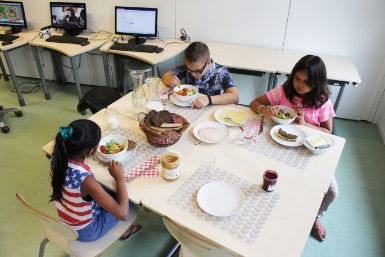 | 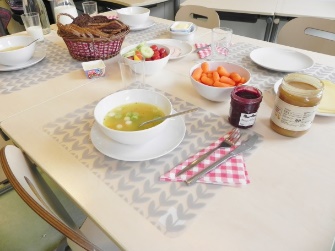 |
| a hot lunch on plates provided at school | Children get every day a hot meal provided by the school. Vegetable snack are also available. The hot meal is brought into the classroom on plates and consists of pasta, rice or potatoes. | 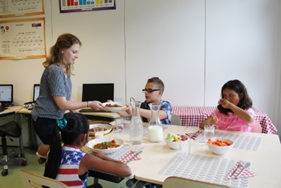 | 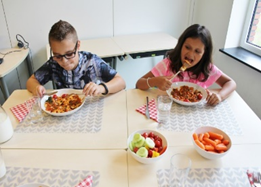 |
| a hot lunch buffet provided at school | Children get every day a hot meal from school. Vegetable snack are also available. The hot meal is brought into the classroom in big pots or pans and consists of pasta, rice or potatoes. Whenever possible children serve themselves and put the food on their plate. | 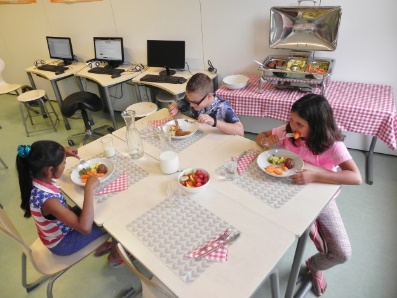 | 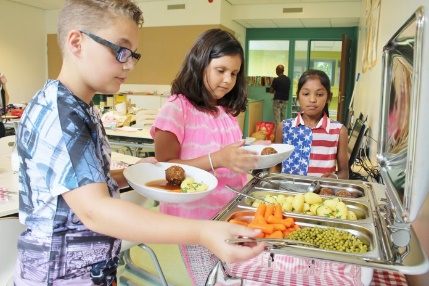 |

**Supplemental 2. Overview of percentages per smiley for sex, educational group and migration background for each school lunch concept.**

|  |  | Red smiley (%) | Orange smiley (%) | Yellow smiley (%) | Light green smiley (%) | Green smiley (%) |
| --- | --- | --- | --- | --- | --- | --- |
| A healthy lunch brought from home | | | | | | |
| All children |  | 8.9 | 12.4 | 26.8 | 30.2 | 21.8 |
| Sex | Male | 9.1 | 12.5 | 28.7 | 25.3 | 24.3 |
|  | Female | 8.8 | 12.3 | 25.1 | 34.4 | 19.5 |
| Group | 5-6 | 7.5 | 9.5 | 25.8 | 32.1 | 25.0 |
|  | 7-8 | 9.8 | 14.2 | 27.4 | 28.9 | 19.6 |
| Migration background | No / Western | 7.5 | 12.7 | 26.0 | 35.1 | 18.8 |
|  | Non-Western | 11.5 | 12.1 | 26.4 | 19.2 | 30.8 |
| Packed sandwiches provided at the school | | | | | | |
| All children |  | 14.7 | 19.2 | 27.2 | 21.4 | 17.4 |
| Sex | Male | 16.9 | 19.9 | 25.7 | 21.6 | 15.9 |
|  | Female | 12.8 | 18.7 | 28.6 | 21.3 | 18.7 |
| Group | 5-6 | 12.7 | 15.1 | 29.4 | 22.6 | 20.2 |
|  | 7-8 | 16.0 | 22.0 | 25.8 | 20.7 | 15.5 |
| Migration background | No / Western | 13.6 | 21.0 | 30.5 | 22.0 | 12.9 |
|  | Non-Western | 14.8 | 16.5 | 20.3 | 19.8 | 28.6 |
| Sandwiches prepared by the children themselves at school | | | | | | |
| All children |  | 7.8 | 11.6 | 26.0 | 28.5 | 26.1 |
| Sex | Male | 11.5 | 11.5 | 28.0 | 25.0 | 24.0 |
|  | Female | 4.7 | 11.7 | 24.2 | 31.5 | 28.0 |
| Group | 5-6 | 7.1 | 9.1 | 27.0 | 25.0 | 31.8 |
|  | 7-8 | 8.3 | 13.2 | 25.3 | 30.8 | 22.5 |
| Migration background | No / Western | 7.7 | 13.6 | 27.8 | 29.6 | 21.3 |
|  | Non-Western | 7.1 | 6.0 | 22.0 | 25.8 | 39.0 |
| Soup or salad with bread provided at school | | | | | | |
| All children |  | 14.2 | 15.0 | 25.4 | 22.4 | 23.0 |
| Sex | Male | 19.3 | 12.8 | 26.4 | 19.3 | 22.3 |
|  | Female | 9.9 | 16.9 | 24.5 | 25.1 | 23.6 |
| Group | 5-6 | 15.9 | 14.7 | 24.6 | 19.4 | 25.4 |
|  | 7-8 | 13.2 | 15.3 | 25.8 | 24.3 | 21.5 |
| Migration background | No / Western | 14.0 | 16.5 | 28.3 | 23.8 | 17.4 |
|  | Non-Western | 14.3 | 11.0 | 18.7 | 20.3 | 35.7 |
| A hot lunch on plates provided at school | | | | | | |
| All children |  | 13.1 | 15.3 | 21.8 | 23.8 | 26.0 |
| Sex | Male | 16.2 | 17.2 | 17.2 | 21.0 | 28.4 |
|  | Female | 10.5 | 13.7 | 25.6 | 26.2 | 23.9 |
| Group | 5-6 | 15.1 | 14.7 | 20.6 | 21.8 | 27.8 |
|  | 7-8 | 11.9 | 15.8 | 22.5 | 25.1 | 24.8 |
| Migration background | No / Western | 14.5 | 18.1 | 21.7 | 25.6 | 20.1 |
|  | Non-Western | 9.3 | 8.8 | 21.4 | 21.4 | 39.0 |
| A hot lunch buffet provided at school | | | | | | |
| All children |  | 12.2 | 13.8 | 20.5 | 19.9 | 33.6 |
| Sex | Male | 14.9 | 13.5 | 18.6 | 18.6 | 34.5 |
|  | Female | 9.9 | 14.0 | 22.2 | 21.0 | 33.0 |
| Group | 5-6 | 13.1 | 13.5 | 18.7 | 15.9 | 38.9 |
|  | 7-8 | 11.6 | 14.0 | 21.7 | 22.5 | 30.2 |
| Background | No / Western | 13.4 | 16.3 | 19.7 | 21.5 | 29.2 |
|  | Non-Western | 8.8 | 8.2 | 23.1 | 15.9 | 44.0 |

| **Supplemental 3.** Results of the ordinal linear regression analyses for the associations between mean support of the alternative school lunch concepts and sex, educational group and migration background. | | | | | | |
| --- | --- | --- | --- | --- | --- | --- |
|  | **A healthy lunch brought from home** | **Packed sandwiches provided at the school** | **Sandwiches prepared by the children themselves at school** | **Soup or salad with bread provided at school** | **A hot lunch on plates provided at school** | **A hot lunch buffet provided at school** |
|  | **OR**§ **+ 95% CI** | **OR**§ **+ 95% CI** | **OR**§ **+ 95% CI** | **OR**§ **+ 95% CI** | **OR**§ **+ 95% CI** | **OR**§ **+ 95% CI** |
| **Sex^a^** |  |  |  |  |  |  |
| Girls | 1.00 Ref. | 1.00 Ref. | 1.00 Ref. | 1.00 Ref. | 1.00 Ref. | 1.00 Ref. |
| Boys | 0.91  (0.75-1.31) | 0.82  (0.63-1.09) | **0.69**  **(0.52-0.91)*** | **0.76**  **(0.57-1.00)*** | 0.90  (0.69-1.19) | 0.93  (0.70-1.22) |
| **Educational group^b^** |  |  |  |  |  |  |
| Group 5-6 | 1.00 Ref. | 1.00 Ref. | 1.00 Ref. | 1.00 Ref. | 1.00 Ref. | 1.00 Ref. |
| Group 7-8 | **0.71**  **(0.53-0.94)*** | **0.71**  **(0.53-0.94)*** | **0.75**  **(0.56-0.99)*** | 1.01  (0.76-1.34) | 1.01  (0.76-1.34) | 0.86  (0.65-1.14) |
| **Migration background^c^** |  |  |  |  |  |  |
| No or a Western migration background | 1.00 Ref. | 1.00 Ref. | 1.00 Ref. | 1.00 Ref. | 1.00 Ref. | 1.00 Ref. |
| Non-Western migration background | 1.06  (0.78-1.44) | **1.60**  **(1.18-2.18)*** | **1.98**  **(1.44-2.71)*** | **1.80**  **(1.32-2.45)*** | **2.11**  **(1.54-2.88)*** | **1.73**  **(1.27-2.37)*** |

Ref, reference category. *P<0.05. §The estimates represent the likelihood to report lower support of alternative school lunch concepts compared with the reference group
